# A Tribute to Mauro Arruda - "A genius of surgery who will be remembered forever"

**DOI:** 10.21470/1678-9741-1-2020-0611

**Published:** 2020

**Authors:** Ricardo C. Lima, Mauro Arruda Filho, Mozart A S Escobar, José Ricardo Lagreca, José Lira Mendes Filho, José Wanderley Neto, José Teles de Mendonça

**Affiliations:** 1 Faculty of Medical Science, University Hospital PROCAPE, University of Pernambuco, Recife, PE, Brazil. E-mail: ricardo.lima@upe.br; 2 Real Hospital Português de Pernambuco Recife, PE, Brazil.; 3 University Hospital PROCAPE, University of Pernambuco, Recife, PE, Brazil.; 4 Federal University of Rio Grande do Norte; Former Director of School Hospital of Federal University of Rio Grande do Norte, Natal, RN, Brazil.; 5 Former Rio Grande do Norte State Secretary of Health, Natal, RN, Brazil.; 6 Federal University of Piauí, Teresina, Piauí, Brazil.; 7 Federal University of Alagoas, Maceio, AL, Brazil.; 8 Instituto de Doenças do Coração, Maceio, AL, Brazil.; 9 Former Alagoas State Secretary of Health, Brazil.; 10 Former President of the Brazilian Society of Cardiovascular Surgery, São Paulo, SP, Brazil.; 11 Federal University of Sergipe; Heart Hospital, Aracaju, SE, Brazil.

Mauro Barbosa Arruda, a pioneer in cardiac surgery, passed away at the age of 89 years on July 21, 2020, at the Real Hospital Português, in Recife, Brazil. It was the same hospital where he implemented a cardiology service of national reference and, in particular, for the Brazilian North-Northeast region. We lost a friend, a teacher, and a surgeon of unique ability.

Born on October 23, 1930, in the state of Pernambuco, he was the third child of four siblings. He started his studies and, at the age of 14, he moved with his family to Recife to finish high school. He needed to find work and to study at the same time, starting at the Companhia Antártica Paulista, in 1947, as a biller, and at the State Health Secretariat in 1948. He joined the Faculty of Medicine of Recife on the following year, just as he started his main life partnership, which lasted 72 years, with Maria Consuelo Monteiro (“Ciêlo”), with whom he built a beautiful family since 1956. This union resulted in two daughters, Flávia and Cláudia, one son, Mauro Filho, six grandchildren, and eight great-grandchildren.

It was in an ebullient scenario, in the 1940s-50s, that the initial milestone of cardiac surgery was established in the world, in Brazil, and especially in the Brazilian Northeast region. In this period, a young doctor stood out: a surgeon who adopted cardiac surgery as his instrument to practice an art. In 1951, at the Hospital Oswaldo Cruz, the birthplace of the University of Pernambuco, the first cardiac surgery in the Brazilian Northeast region was performed by the imminent surgeon Joaquim Cavalcanti, assisted by the young doctor Mauro Arruda. Thus, a close connection was created between his professional life and cardiothoracic surgery. With a unique zest, a surgeon began to emerge, who later would surpass the limits of just being a good surgeon and start to occupy the spot reserved for creators. With a surgical skill rarely observed and recognized by all, Dr. Mauro managed to transform what seemed to be almost impossible into resolved situations. A real genius! That was Mauro Arruda.

## First Contact with the Heart

Still as a student at the end of the 4th year of medical school, in 1952, Dr. Mauro went to the Hospital Oswaldo Cruz to meet Professor Joaquim Cavalcanti, who soon welcomed him as a member of the team, and thus his relationship with surgery began. Immediately and excited about the art of surgery, he started his internship at the thoracic surgery service. He realized that it was a unique, pleasant environment, where cordiality and seriousness reigned. During his medical internship, Mauro got to know an environment where he learned that medicine could be practiced with no financial interest, treating wealthy and poor people in the same way. After receiving his medical degree from the Faculty of Medicine of the University of Recife, in December 1954, he continued in the service and participated in the construction of the Malaquias Gonçalves Pavilion, which became the birthplace of cardiac surgery in the Brazilian North-Northeast region. He participated directly in the first mitral commissurotomy and the first systemic pulmonary shunt in the state of Pernambuco. The first child operated on by Prof. Joaquim, diagnosed with tetralogy of Fallot, underwent a Blalock-Taussig anastomosis, with Mauro as one of the main assistants in the procedure. It was at that moment that he established his name as one of the pioneers in the history of cardiovascular surgery in Brazil. During the period from 1952 to 1956, initially as a doctorate student and then as a physician, he had the opportunity to learn the art of cardiothoracic surgery. He actively participated in the initial development of Brazilian cardiac surgery until 1956, when the thoracic surgery in Pernambuco was orphaned with the death of its mentor Joaquim Cavalcanti.

During the Brazilian Congress of Cardiology in Recife, in 1956, Dr. Mauro presented two papers: "Tetralogy of Fallot, clinical evolution of five operated cases" and "On a case of pulmonary arteriovenous fistula and concomitant mitral stenosis". This congress was attended by Prof. E J Zerbini to whom clinical cases of possible surgical resolution were presented, including a mediastinal tumor. Prof. Zerbini requested an angiography and the young Dr. Mauro immediately offered himself to take on that challenge at the time. The examination, done by hand, revealed a brachycephalic trunk aneurysm, successfully operated by Prof. Zerbini at Hospital Otávio de Freitas. Back in São Paulo, Prof. Zerbini, after countless compliments to Mauro Arruda, invites him to be a fellow in his unit at the Hospital das Clínicas of the University of São Paulo Medical School with grants of Capes and Rockefeller Foundation.

In 1957, he began his experience with Prof. Zerbini, becoming his first resident physician, actively participating in the beginnings of open-heart surgery at the University of São Paulo Medical School. At that time, cardiopulmonary bypass (CPB) was performed on dogs in the experimental surgical laboratory of Hospital das Clínicas and there was an intense participation of the young fellow. This period was remarkable in the formation of his medical personality because, in addition to learning with the exceptional surgical quality from Prof. Zerbini, Dr. Mauro also learned from his enormous capacity for work and remarkable human qualities. Dr. Mauro participated in the expansion of cardiac surgery in Brazil and South America. He also participated in the creation of a homologous and heterologous arterial grafts bank, with grafts preserved in 96% alcohol, the Artery Bank of the University of São Paulo Medical School.

On his return to Recife, he fulfilled his commitment as a fellow with Capes and Rockefeller Foundation, starting as a researcher at the Institute of Cardiology of Hospital Dom Pedro II, this time under the guidance of Prof. Luiz Tavares. The objective was to establish open-heart surgery and, in parallel, Dr. Mauro assumed the position of thoracic surgeon at Hospital Oswaldo Cruz, and then at Hospital Otávio de Freitas.

At the Institute of Cardiology, Dr. Mauro began following the model of the University of São Paulo Medical School, with initial training of the entire team on the new surgical model and the creation of an experimental surgical unit. On April 11, 1960, the first atrial septal defect was successfully corrected, with the aid of CPB, with Luiz Tavares and Mauro Arruda as main surgeons. One year later, the first surgery to correct a ventricular septal defect with deep hypothermia and total circulatory arrest for 32 minutes was performed for the first time. After this achievement, the Institute of Cardiology became an active, productive center, responsible for the training of countless clinical cardiologists and surgeons, carrying out and publishing research.

In the years 1962-63, Dr. Mauro was awarded a grant from the Coopération Technique Internationale for an internship with Prof. Charles Dubost at Hospital Broussais and Hospital Marie Lannelongue, in Paris. He had active and intense participation in coronary surgery, in the implantation of mitral and aortic prostheses, and the surgical treatment of congenital heart diseases. Prof. Dubost asked him to stay in Paris, but Dr. Mauro preferred to return to Recife to carry out the work he had begun at Hospital Oswaldo Cruz and the Institute of Cardiology.

### Chest Diseases Unit

With the creation of departments in Brazilian federal universities by the military government, there was a natural extinction of the Institute of Cardiology. By that time, and with a group of doctors, Dr. Mauro created the Chest Diseases Unit at Hospital Barão de Lucena, inaugurated in the presence of Prof. Zerbini. However, to the group's surprise, the hospital was sold to the extinct National Institute of Medical Assistance for Social Security (Inamps). In the short period between August 1968 and August 1971, 216 patients, 170 under CPB, were operated on. Dr. Mauro had the opportunity to implant a pioneering autologous biological fascia lata graft in Brazil in an aortic and mitral position. These prostheses were mounted on a type of ring brought from England and one of these was given to Prof. Zerbini as a gift. The ring was reproduced in the workshop of the Hospital das Clínicas of the University of São Paulo and a new biological prosthesis was created using dura mater as the biological tissue of the leaflets. These valves were made available in Brazil and Latin America, benefiting thousands of patients at a very low cost.

### Institute of Chest Diseases of Recife

With the sale of the Hospital Barão de Lucena to Inamps, it was impossible for the Chest Diseases Unit to remain in that institution. Under the leadership of Mauro Arruda, the group transferred to the Hospital Português de Beneficência de Pernambuco, starting the Institute of Chest Diseases of Recife (IDTR), formed by four partners and several collaborators. This service led the hospital to the path of progress and treatment of patients with high complexity. In February 1973, IDTR acquired the first hemodynamic monitoring device in the hospital and the first cinecoronariography in the Brazilian Northeast region was performed. Then, the first coronary artery bypass surgery with saphenous vein grafts and a new coronary artery bypass surgery using the mammary artery were performed. These accomplishments led to a great stimulus to the development of cardiac surgery and cardiology in the region. Several professionals originated from this initial nucleus at IDTR - Real Hospital Português de Beneficência in Pernambuco, but IDTR, unfortunately, ended its activities in 1986.

### Cardiovascular and Thoracic Surgery Service

To continue his art, Dr. Mauro created another service whose team base was his family nucleus, formed by Mauro himself and his children Mauro Arruda Filho, Flavia Arruda, and Claudia Arruda, in addition to other collaborators. He extended cardiac surgery in the city of Recife with the creation of the Institute of Medicine and Surgery (Imec) and was a pioneer in taking cardiac surgery to areas outside the state capital, with the creation of the first cardiac surgery service in Caruaru, at Hospital Santa Efigênia. The Real Hospital Português de Beneficência and Caruaru surgery services are currently active, under the leadership of his son, the cardiovascular surgeon Mauro Barbosa Arruda Filho. During its entire period of surgical activity, 22,971 cardiothoracic surgeries were performed.

### Pioneering

Dr. Mauro was a surgical pioneer, in the state of Pernambuco and the Brazilian North-Northeast region, regarding CPB in 1960 (atrial septal defect), correction of a ventricular septal defect with deep hypothermia in 1961, implantation of an endocardial pacemaker, epicardial pacemaker, and mitral and aortic valve surgery in 1970, myocardial revascularization surgery with the saphenous vein in 1971, and with an internal thoracic artery in 1973, as well as the introduction of myocardial protection in 1977.

### An Itinerant Surgeon

At that time, most inland cities and even some capitals in Brazil suffered from the lack of cardiology services and cardiac surgery. There were no pacemakers for sale in Brazil and the devices were purchased abroad, with active participation from Varig Airlines. Dr. Mauro performed surgeries in different locations in the Northeast region. He implanted the first cardiac pacemaker in Campina Grande (Paraíba). In 1969, in Aracaju (Sergipe), he performed two pioneering surgeries in the state, a ligation of the arterial duct and a pacemaker implantation, among many other procedures.

### An Innovative Surgeon

With his innovative side, Dr. Mauro left, among many others, two major contributions to cardiac surgery, specifically in the surgical treatment of the mitral valve. He devised a biological valve model and an innovative technical variation for the preservation of the mitral valve.

Dr. Mauro insisted that, when replacing a mitral valve with a circular prosthetic ring, it caused a change in the shape of the natural ring, with consequences on the geometry of the left ventricle and he believed that the valve should be as similar as possible to its native shape, to better prevent ventricular dysfunction. He created an ellipsoid mitral prosthetic ring that more closely resembled the natural mitral valve ring. The ring had an elliptical shape and only two leaflets, very similar to the native mitral valve. In this way, he operated on several patients with excellent immediate results, being published in the Journal of the Brazilian College of Surgeons.

Due to his experience with valve lesions caused by rheumatic fever, especially mitral insufficiency in children and adolescents, and limitations in the use of biological prosthesis with early degeneration, he devised a technical variation of mitral valve repair, the “concentric mitral ring annuloplasty”, in 1979. The technique made it possible to treat countless children and adolescents with the purpose of stabilizing the functional mechanism of the mitral valve.

### Academic and Associative Life

Dr. Mauro started his academic life as an assistant professor at the 1st Chair of Surgical Clinic at the Faculty of Medical Sciences, in 1955, and later at the Federal University of Pernambuco (UFPE), in the discipline of thoracic surgery, as an assistant professor, adjunct professor, associate professor, and regent professor of the discipline of thoracic surgery in the master's degree course.

In his associative life at the Brazilian Society of Cardiovascular Surgery (SBCCV), he was a founding member, a full member, and a redeemed member. He was also a cardiovascular surgery specialist by the Brazilian Medical Association (AMB), redeemed member of the Brazilian Society of Cardiology, emeritus member of the Brazilian College of Surgeons, president of the Society of Cardiology of Pernambuco state (1972-1973), and founding member, full member, and president of the North-Northeast Society of Cardiovascular Surgery (1998-1999).

Mauro Arruda's legacy is very rich. He participated in thousands of surgeries and trained and influenced hundreds of cardiologists, clinicians, and surgeons, many of whom very successful in their cities through a seed planted at the Hospital Oswaldo Cruz, followed by the Institute of Cardiology - Hospital Dom Pedro II, which resulted in the development of the largest medical center of cardiology in the Brazilian Northeast region, the Real Hospital Português de Beneficência in Pernambuco.

### Testimonials

#### Ricardo Lagreca

“With his origins in the school of a master - Joaquim Cavalcanti -, Dr. Mauro developed with him the first steps of a surgeon who, later on, would occupy a place among the immortals. The new world of cardiac surgery that he saw born dazzled him and his ability with his hands would become a major factor in reducing the subjectivities and natural difficulties of a time that was still beginning as a true technological revolution. It was not difficult for everyone to learn about him, his qualities, his posture as a natural leader in the use of hands, and to fully integrate him in what would become the mainstay for the development of cardiac surgery in the Northeastern region and, why not say, in Brazil. From all corners of the country, he attracted people who saw in him the hope of curing their illnesses. He will always be remembered as a genius of skillful hands, as of other geniuses of other arts. A real genius!”

#### Mozart Escobar

“I met Mauro Arruda by a friend of my father. I told him: ‘I would like to join your service’. At this moment, my father's friend asked me what year in college was I in, and I embarrassedly said I was a sophomore. This ‘friend’ immediately asked me if it was not too early to do an internship. I tried to justify the precociousness when Dr. Mauro told me something that I never forgot and took as a way of life: ‘It is never too late to learn and never too early to start’. This was Dr. Mauro Arruda, my professor and friend during these 52 years in which he gave me the chance to be around him, inside and outside the operating rooms.”

#### Ricardo Lima

“Mauro Arruda was the inspiration I needed to decide to donate my life to cardiac surgery. In a class on the course he taught, I was sure that my vagueness was resolved. In the next moment, I did an internship in the 2nd Surgical Clinic of Thoracic Surgery at Hospital de Clínicas Dom Pedro II, and since then I have never been away from cardiac surgery and from Mauro. For 45 years, I was his student, doctoral student, resident, assistant, colleague, and friend. What made him a born leader was a set of qualities such as simplicity, humanity, honesty, ethics, solidarity, excellent humor, and common sense. As a surgeon, he was brilliant, easily transforming difficulties in possibilities. He used his surgical quality like few others, and I can say, in my modest experience as a surgeon, I did not know a thoracic and heart surgeon of such quality. He was unique and I am just grateful for the opportunities!”

#### José Lira Mendes Filho

“Life, with its elusive character, usually leaves us marked by longing. Now, this longing has a name: Mauro Arruda. Especially for me, his eternal apprentice, with whom he has built a friendship since 1978 and has shared many of the good, and also not so good, moments of life as an experienced, skilled, intelligent, and intellectually restless surgeon. I tried hard to copy, besides his operative technique, his ethical integrity, of clear opinions, without factionalism. During our 41-year relationship, we became friends and pleasant brotherly pranks were always present at our meetings. As a surgeon, a lot of what I am I got from him, and on several occasions, I have had the pleasure of thanking him. But there were still few acknowledgments, which will now turn into memories, stories, and longing. My dear boss and friend Mauro Arruda precedes us in eternity, immortalized.”

#### Mauro Arruda Filho

Over the past 56 years, I have had the privilege of living intensely with my dear friend, master, and beloved father, Mauro Arruda. From the boy in short pants who wandered the slopes of Bom Jardim, rode through the floodplains of the Tracunhaém river and the red clay fields of Surubim, to the pioneer professional, with unparalleled skill and irreproachable ethical formation, and to the exemplary father and friend, I had the opportunity to share so many moments along his trajectory, which provided me, along with Ciêlo's guidance, a solid personal and professional training.

Mirrored in this experience, I try to transmit to my children the teachings received through example, dialogue, justice, and love. In the art of healing, even without the same skill, I seek to follow the path of ethics and, above all, the well-being of others, the essence of everything.

To paraphrase Bernard Shaw, “there are men who see things as they are and simply wonder ‘why?’. I see things as they should be, and I wonder ‘why not?’”. My father was one of these predestined and enlightened people who managed to see beyond his time, always returning to Pernambuco, and unselfishly, following the example of his masters Joaquim Cavalcanti, Zerbini, Dubost, and Luis Tavares, contributed to the training of so many professionals, thus leading cardiac surgery in the Northeast region to a distinguished level.

Today I just miss him, but I am thankful to God for the privilege of having been guided in the life teachings and my professional training by a great Friend, Master, and beloved Father. See you soon… God Save Maurão!”

#### Edgar Guimarães Victor

“Particularly considering that his aspiration was directed at the very closed and recently implanted cardiac surgery, the young surgeon used two traits that were natural to him: character and talent. Mauro Arruda was a surgeon who solved problems, regardless of the recognition of personal merit or the award of laurels. He shared his knowledge and skills with colleagues, veterans, and newbies. Humming or whistling softly during the hours of greatest tension in the surgical field, agile, absolutely cold at work - he carried tons of emotion with him. Character and talent: these are Mauro Arruda’s legacies.”

#### José Wanderley Neto

“He was a pioneer, of the first generation of cardiovascular surgeons. A complete surgeon, a professor, and an undisputed leader. I met him as soon as I returned to Maceió, in 1978. It was love at first sight. The age difference of 20 years was not a reason for distancing. He integrated himself loosely into the expansion movement of cardiac surgery among us with the joviality of a privileged head, typical of men of great wisdom. Throughout all these years, we participated in many scientific journeys, in homage to him, and we shared leisure moments and long conversations about life, accompanied by a good scotch. At those times, we were all at the same level. It was when the human figure was unveiled and showed all his greatness. It is rare to find men like that and even more to have the privilege of being close to them. I will miss him. Mauro left a legacy between us. A big hug to you, Mauro. Any day we will see each other.”

#### José Teles de Mendonça

*“I met Prof. Mauro Arruda in 1969 when he pioneered the first cardiovascular procedures in Sergipe. I was entering the School of Medicine and, from the display, I watched the surgical spectacle from the amphitheater in room III of the Hospital de Cirurgia. The Master, at that moment, enchanted me by his operative skills. Then, in the more than 50 years that I was able to enjoy his company, he definitely won me over with his permanent lessons of wisdom. He was a genius. His life, an infallible enchantment machine. His actions constantly sought perfection and the example indelibly marked everyone who had the privilege of approaching him. Rest in peace, beloved master.*”

## Figures and Tables

**Figure:**
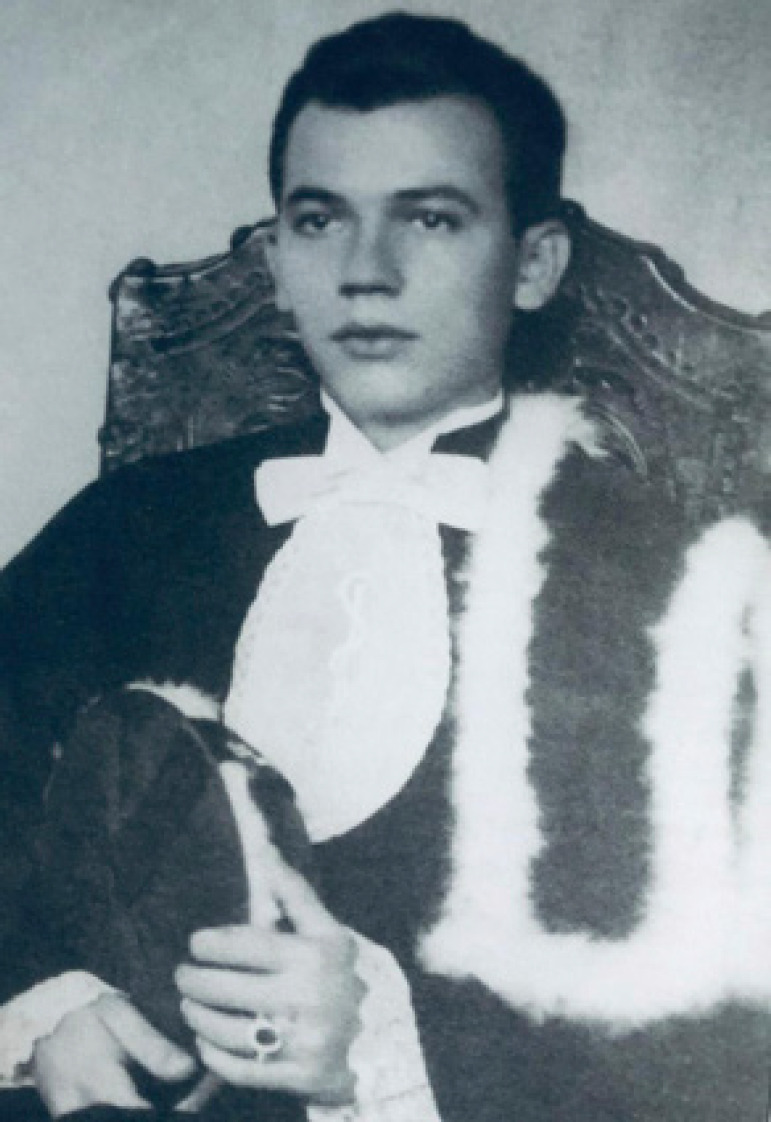
Professor Mauro Arruda receiving his medical degree in 1954.

**Figure:**
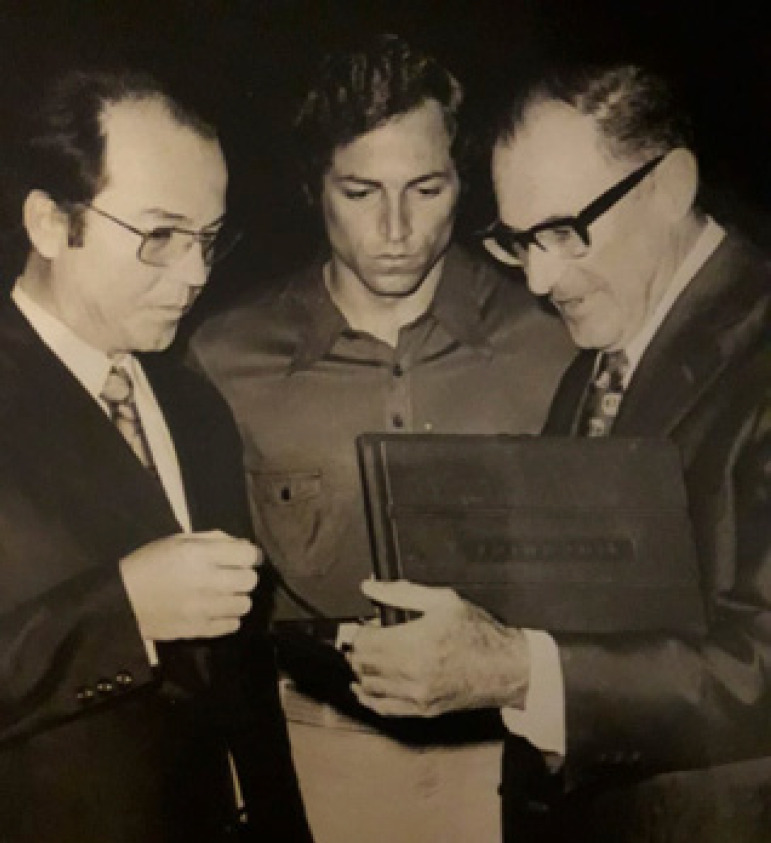
Professors Mauro Arruda, Mozart Escobar, and E J Zerbini in a Scientific Meeting, Recife 1972.

**Figure:**
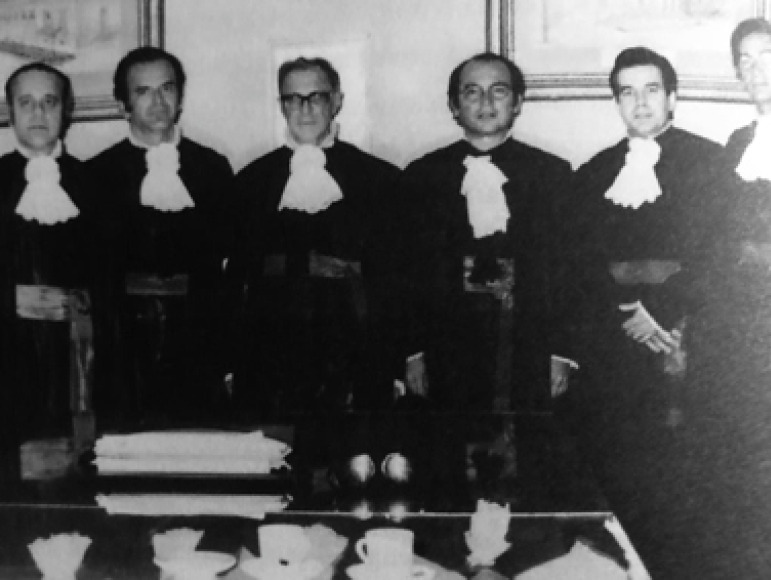
Examination Board of the Associate Professor Otoni Moreira Gomes. From left to right: Professors Izeu Afonso da Costa, Marcelo Campos Cristo, E J Zerbini, Mauro Arruda, Otoni Moreira Gomes, and Geraldo Virginelli.

**Figure:**
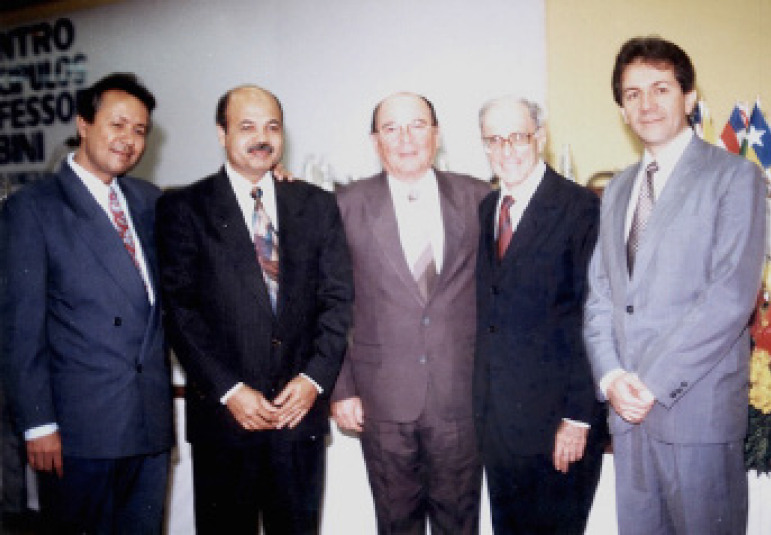
Nort-Northeast Cardiovascular Surgery Meeting and Disciple of Prof. Zerbini Meeting, 1992. From left to right: Jose Wanderley, Jose Teles, Mauro Arruda, E J Zerbini, and Ricardo Lima.

**Figure:**
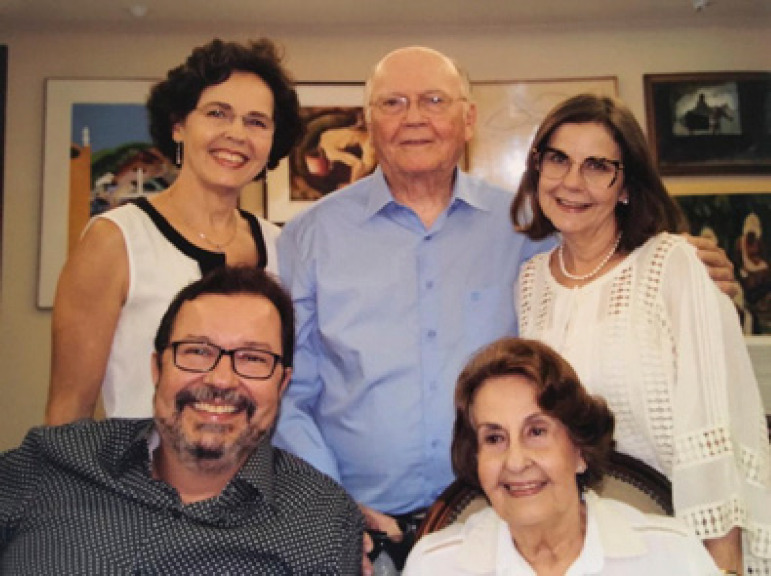
Mauro Arruda with his family: Maria Consuelo, Claudia, Flavia, and Mauro Filho, 2016.

**Figure:**
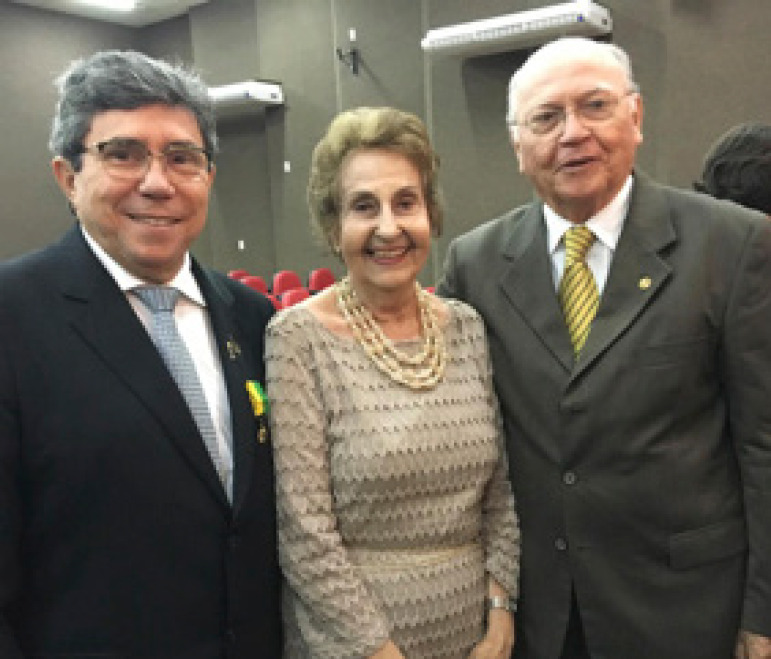
Professor Mauro with Consuelo and Professor Jose Lira, his former resident doctor, 2016.

